# Toward a dual process model of moral injury and traumatic illness

**DOI:** 10.3389/fpsyt.2022.883338

**Published:** 2022-08-24

**Authors:** Nicholas Barr, Hazel Atuel, Shaddy Saba, Carl A. Castro

**Affiliations:** ^1^School of Social Work, University of Nevada, Las Vegas, Las Vegas, NV, United States; ^2^Suzanne Dworak-Peck School of Social Work, University of Southern California, Los Angeles, CA, United States; ^3^Center for Innovation and Research on Veterans and Military Families, University of Southern California, Los Angeles, CA, United States

**Keywords:** posttraumatic stress disorder (PTSD), veterans, trauma, stress, moral injury

## Abstract

Moral injury has emerged as a topic of significant research and clinical interest over the last decade. However, much work remains to be done to comprehensively define the moral injury construct, with implications for understanding the etiology and maintenance of moral injury, its symptoms, associations with and distinctions from traumatic illness, and treatment approaches. We provide a brief overview of the existing moral injury literature and introduce a novel dual process model (DPM) of moral injury and traumatic illness. The DPM posits an event exposure which may satisfy DSM-5 posttraumatic stress disorder (PTSD) criterion A, potential morally injurious event (PMIE) criteria, or both, followed by individual role appraisal as a perpetrator through action or inaction, a witness, a victim, or a combination of the these. Role appraisal influences symptoms and processes across biological, psychological, behavioral, social, spiritual/religious, as well as values, character, and identity domains to support a label of traumatic illness, moral injury, or both. The DPM provides a flexible analytical framework for evaluating symptoms associated with moral injury and traumatic stress and has important implications for treatment. The most thoroughly reviewed evidence-based interventions for traumatic stress hinge on exposure and habituation mechanisms to manage dysregulation of fear and memory systems, but these mechanisms often do not address core domains of moral injury identified in the DPM, including spiritual, religious, values, character, and identity domains as these exist largely outside of the putative fear network. We provide brief vignettes to illustrate the practical application of the DPM and argue that adjunct and stand-alone approaches which address values and character domains, leveraging principles of Stoicism, non-judgment of experience, acceptance, and values-oriented action, are more likely than traditional trauma treatment approaches to positively affect moral injury symptoms.

## Introduction

The dual process model of moral injury and traumatic illness traces a four-stage evaluative framework by which event-exposures lead to traumatic illness, moral injury, or both. A final, fifth stage sketches intervention and healing approaches targeted toward, in the case of traumatic illness, classical exposure-habituation models to regulate disruptions in fear and memory processing and, in the case of moral injury, novel approaches to enhance acceptance, facilitate cognitive and emotional flexibility, and develop meaning to heal disruptions in religious/spiritual and identity, values, and character domains. Here and throughout the text, we use the term “traumatic illness” to refer to the family of trauma and stressor related psychological disorders associated with trauma experiences, the most well-known of which is posttraumatic stress disorder [PTSD; (1)].

### Defining moral injury

Military service members and veterans can suffer an exacting physical and psychological toll from combat (2). Particularly well established are the negative health effects of traumatic combat stressors, which involve the experience or threat of serious injury or death and can result in PTSD (1, 3). Several rich psychological theories have been developed in recent decades to elucidate the pathways through which traumatic stressors can lead to the development of traumatic illness, particularly PTSD (4). The most prominent of these theories, such as the Emotional Processing Theory and the Cognitive Theory of PTSD (5, 6), investigate the effects of traumatic stressors on an individual’s memory and fear processing systems as well as beliefs about safety and personal agency in the world. Importantly, as these theoretical models of PTSD inform prominent interventions targeting psychologically distressed veterans (7, 8), the efficacy of these interventions may be limited if models do not accurately reflect the range of combat-related stressors and their heterogeneous impacts.

In relatively recent years, practitioners and researchers working with military and veteran populations have investigated not only psychological but *moral* distress that can result from profoundly disturbing experiences, and they have dubbed such distress *moral injury* (9). The earliest descriptions of moral injury are typically attributed to United States Department of Veterans Affairs (VA) psychiatrist and researcher Shay (10), who first conceptualized moral injury while studying Homer’s *Iliad.* Shay’s early conceptions of moral injury focused on the impact of moral failures by those in authority in the context of the Vietnam War; he drew parallels between experiences of betrayal and their consequences depicted in the *Iliad* and the effects of failures of United States leadership on service members in Vietnam. Subsequently, Litz et al. (11) broadened the study of moral injury to focus more deeply not just on the individual moral consequences of the failures of trusted others but also on moral transgressions committed by individuals themselves (e.g., killing a non-combatant) and moral failures by inaction (e.g., failing to prevent disproportionate violence). While studies of moral injury have since proliferated and the field has expanded to incorporate the work of scholars from a wide range of medical, behavioral, and social science disciplines, the field is still relatively nascent and fundamental conceptual questions require investigation and clarification (9).

Specifically, as researchers have sought to develop a nuanced understanding of moral injury, there is a need to further clarify both the types of events (i.e., moral stressors) that can lead to moral injury and the effects that those experiences can have on an individual [i.e., the resulting distress, or moral injury outcomes; (9, 12)]. There is a parallel to be drawn here with the study of traumatic stress and the distinction between traumatic events (i.e., fearing for one’s life during combat) and the potential effects of such events (i.e., PTSD). With respect to moral injury, precipitating events have been called *potentially morally injurious experiences* [PMIEs; (13)]. Conceptions of what constitutes PMIEs are still developing. Shay, for example, came to define a PMIE as a betrayal of justice by a person in authority in a high-stakes situation (14). Litz’s et al. (15) definition, which has gained prominence, defines PMIEs as experiences that involve “perpetuating, failing to prevent, bearing witness, or learning about acts that transgress deeply held moral beliefs and expectations” (p. 697). While contemporary empirical studies of PMIEs tend to incorporate elements of both Shay’s (i.e., betrayal) and Litz et al.’s conceptions (i.e., perpetuating or witnessing transgressions), efforts to determine the validity of PMIE constructs are still ongoing (9, 13). Notably, as is implied by the phrase “*potentially* morally injurious experiences,” not everyone who experiences a PMIE goes on to develop moral injury.

Additionally, efforts to characterize the effects of PMIEs on individuals who *do* go on to develop moral injury (i.e., moral injury outcomes) are also ongoing. Shay [(16); p. 26] regarded the *Iliad* as “the story of the undoing of Achilles’ character” and likewise believed the veterans with moral injury whom he treated had developed a character wound as a result of their experiences (14). However, most prominent conceptions of moral injury have been developed by clinician-researchers who have tended to narrow the scope and focus on sequelae that typically fall within the clinical purview (17). For example, Litz and colleagues (15) cite PTSD symptoms, difficult emotions (e.g., shame, anxiety, and hopelessness), and self-harming and self-handicapping behaviors (e.g., suicidality and substance use) as several defining features of moral injury. Researchers have since linked PMIE exposure to various physical (18), behavioral health (19), sociocultural (20, 21), and spiritual (22) outcomes. However, there remains a fundamental lack of theoretical and empirical work investigating the role of values, character, and identity in the development of moral injury and its consequences despite the central importance of character in Shay’s original conceptualization. Such an investigation is critically important for two key reasons: first, to delineate the contours of the moral injury construct and identify areas of overlap and distinction from traumatic illness, and second, to identify potential symptom and process domains which must be targeted to facilitate moral healing. If what is harmed in moral injury is, at least in part, character and moral identity derived from moral values, the role of these domains in the experience of moral injury must be understood so they may be addressed in a healing process.

## A dual process model of moral injury and traumatic illness

**FIGURE 1:**
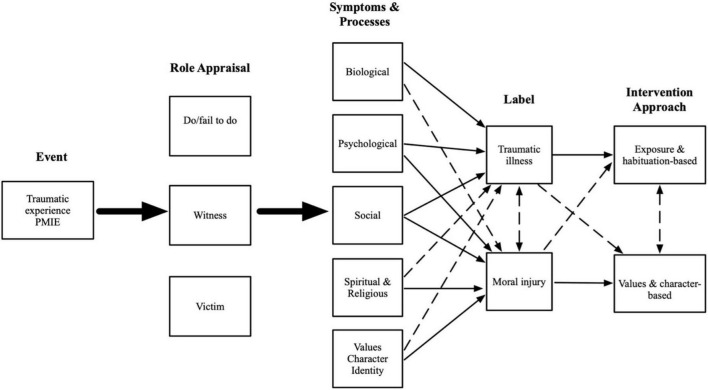
Conceptual diagram of the dual process model of moral injury and traumatic injury. The two large solid arrows indicate that events can lead to any and all role appraisal, and that any and all role appraisals can lead to any and all symptoms and processes. Thin solid arrows indicate a primary/strong link between individual symptoms and processes and labels and intervention approaches. Thin dashed arrows indicate a secondary/weak link between individual symptoms and processes and labels and intervention approaches.

To better understand pathways leading from (1) adverse experiences, including PMIEs and traumatic stressors, to (2) role appraisals and (3) associated symptoms and processes, to (4) useful diagnostic or descriptive labels, and (5) approaches to intervention and healing, we propose a dual process conceptual model of moral injury and traumatic illness. Under the dual process model (DPM) framework, we explore the pathways through which moral failures can result in a pattern of experience best characterized by moral injury, both alongside and in contrast to the pathways through which traumatic events can lead to traumatic injury and traumatic illness. We argue that while both moral injury and traumatic illness can follow from discrete events or the same single event and can exist either alone or simultaneously (e.g., in a comorbid fashion), their developmental pathways are best characterized by different symptom and process domains, resulting in a continuum of subjective experience characterized by varying intensity of moral injury and traumatic illness. Finally, we explore implications of the DPM for intervention and healing in the context of moral injury. Figure 1 provides an overview of the DPM.

### Event-exposure

The event-exposure stage of the dual process model refers to a consequential initial event (or accumulation of experiences) consistent with the PTSD criterion A of the *Diagnostic and Statistical Manual of Mental Disorders* [5th ed.; DSM–5; (1)], or the definition of a PMIE (9, 13), or both. Examples of traumatic experiences abound in the literature, from physical and sexual violence to combat in wartime to natural disaster. Our goal at this stage is not to provide an exhaustive list of traumatic experiences but to signal a broad set of events generally understood in the clinical literature to provoke intense and overwhelming emotion that disrupts normative emotion regulation and memory encoding processes (5, 6). Classically, experiences which satisfy DSM criterion A are characterized by the emotions like fear and shame (DSM-5), but this need not be the case in order for the classical PTSD processes to develop. Consistent with previous accounts of moral injury (11, 12), we argue that an event-exposure may be a PMIE if it involves a meaningful moral transgression, defined as a violation of closely held moral values or beliefs, with salient consequences (11). As is the case with PTSD, expression of specific symptoms and processes due to the event-exposure is required in order to make a determination about whether a PMIE is in fact a morally injurious event (12). In other words, moral injury is identified by exploring domains of human experience over time and not by discrete events. Understanding the interaction between an event-exposure, the individual’s appraisal of their role in the event, and the nature of the biological, psychological, social, spiritual/religious, and values, character and identity symptoms and processes that follow is required in order to appropriately determine whether an individual is experiencing traumatic illness, moral injury, or both.

### Role appraisal

Researchers concerned with moral injury have developed a taxonomy of individual role appraisals following exposure to adverse experiences (i.e., traumatic events or PMIEs) corresponding to perpetrator, witness, and victim, though these appraisals are dynamic and may overlap and change with time (11, 17). In the context of a PMIE, the perpetrator role is often characterized by acting or failing to act in the context of a morally transgressive event. The perpetrator appraisal, whether through an act of commission or omission (23) burdens the individual with a sense of personal responsibility for moral failure. The individual’s character, identity, and narrative of their own morality and values is called into question (17). The witness role is defined by the individual’s direct experience of another’s moral transgression, but one in which they do not view themselves as having had the power to intervene. The witness appraisal, in distinction to the perpetrator appraisal, locates the moral transgression at the heart of a PMIE externally — rather than destabilizing internal moral architecture, the witnesses’ external moral framework, characterized by faith in important others (e.g., leaders, colleagues), rules and institutions, even society more broadly, is destabilized. The victim appraisal, like the witness appraisal, locates the responsibility for moral transgression at the heart of a PMIE externally. The victim appraisal may confer a more acute sense of personal violation and betrayal, destabilizing externally located moral frameworks and, potentially, confidence in moral judgment about trusted others.

Role appraisal influences symptoms and processes across biological, psychological, behavioral, social, spiritual/religious, and, consistent with Shay’s (24) account, values, identity, and character domains. In general, it is expected that perpetrator appraisal activates internally directed symptoms and processes like shame, guilt, and crises of moral identity and values, whereas witness or victim appraisals activate externally directed symptoms and processes like betrayal, anger, and crises of faith in external structures of morality (12). While we have described role appraisal in the context of a PMIE, appraisal also has a role in the development and maintenance of traumatic illness. In this context, appraisal typically describes evaluation of threat and danger, and problematic appraisals reflect deficient integration of traumatic memories leading to intrusive thoughts and attendant emotional and physiological dysregulation (25–27). Thus, while individuals may certainly identify themselves as perpetrators, witnesses, and victims in the context of traumatic experiences, the literature tends to characterize appraisal in the context of traumatic illness as evaluating the dangerous event and the dangerous world; in the context of moral injury, appraisal evaluates the moral self and the standard-bearers of the moral order.

Finally, we argue that moral injury may compel an individual to take on non-discrete (e.g., overlapping) roles as a function of *space* and *time* (17). Briefly, space is simply the physical location of a given PMIE. Within a particular space, an individual can begin in one role (e.g., witnessing a battle buddy die) and can potentially take on other roles as they move within that same space (e.g., perpetrator in retaliatory act). In the aftermath of an PMIE, time moves an individual to take on an additional role: that of a witness. This is because the rumination process compels an individual to bear witness to the event. Regardless of an individual’s initial role(s) (perpetrator, victim, and/or witness), memories serve a forcing function of situating an individual as an actor-observer of the same event.

### Symptoms and processes

The DPM posits five symptom and process domains that can be examined and applied to individual experiences to produce a profile conditioned on the role appraisal that follows a PMIE or traumatic experience. These five domains are: biological, psychological, social, spiritual/religious, and values, identity and character. A strength of the dual processes model is its ability to account for the complex interplay of symptoms and processes across these domains to support a best-fit profile consistent with traumatic illness, moral injury, or both.

#### Biological domain

While moral injury has emerged as an important topic in the psychological and behavioral health literature, investigators and clinicians are still working toward identifying biological markers and other indicators associated with moral injury. In contrast, there has been enormous interest in refining biological accounts of traumatic illness since the early 2000s (28, 29). While these accounts must, like other perspectives, grapple with the enormous heterogeneity of symptom presentations under the traumatic illness framework, biological studies of traumatic illness and PTSD in particular have highlighted several properties of the disorder, including alterations in neuroendocrine system function (e.g., low cortisol and high epinephrine levels, higher autonomic response following exposure to trauma cues) and alterations in brain structure (e.g., reduced hippocampal and cortical volume) associated with PTSD diagnosis (30, 31). Biologically driven investigations of PTSD have also highlighted the interplay between genetic risk factors, environmental exposures, and epigenetic processes that may undergird risk for the development of PTSD following exposure to traumatic experiences (32).

While contemporary descriptions of traumatic illness and PTSD have moved beyond relatively narrow fear and memory-based sequelae following exposure to life-threatening events or sexual assault, biological accounts nevertheless highlight the important role of systems and structures associated with fear, stress, arousal, memory, and learning in the etiology and maintenance of PTSD (28, 33). When the physiological expressions of these processes are evident in the context of a traumatic event exposure and additional psychological symptoms strongly associated with traumatic illness, the presence of traumatic illness is strongly indicated. Conversely, we argue that moral injury is better defined, at least for now, by non-biological domains. Furthermore, strong evidence has yet to connect symptoms like hyperarousal and reexperiencing, which are amenable to biological explanation of threat-response system disruption, to PMIEs and their sequelae, leading some researchers to hypothesize that moral injury may be mediated by pathways distinct from threat-based traumatic illness (34, 15).

#### Psychological domain

While trauma and stressor-related disorders comprise an entire diagnostic cluster in the DSM 5 (2013), PTSD remains the core expression of traumatic illness. PTSD is a psychological disorder and is recognized and understood by its four symptom clusters: reexperiencing symptoms, avoidance symptoms, alterations in arousal and reactivity, and with the introduction of DSM-5 (2013), negative alterations in mood and cognition. The reorganization of PTSD criteria in DSM-5 marks a significant conceptual overhaul of the disorder. The first indicator of this reconceptualization is the transplanting of PTSD from anxiety disorders category into its new category, trauma and stressor-related disorders. Second, the addition of the negative cognitions and mood symptom cluster made possible over 600,000 possible PTSD diagnostic combinations which allow an individual to meet criteria for the disorder (35) – 52% of these do not contain any symptom overlap. These changes reflect a broadening of the universe of symptoms which might characterize PTSD such that two individuals can have symptom profiles with zero overlap that both meet diagnostic criteria for the disorder (32, 36). While a larger debate about the epistemological and clinical value of these changes is beyond the scope of this manuscript, it seems plausible that less well-developed domains of human experience, like moral transgression, moral failure, and moral injury, may have been subsumed under the PTSD rubric to account for things that seem like PTSD but may reflect less well-understood patterns of symptoms and processes. Certain psychological symptoms (e.g., nightmares reliving terrifying experiences, avoidance of fear-inducing reminders of traumatic experiences) seem to function as cleaner indicators of a narrow PTSD diagnosis. However, scholars of moral injury have argued that it is plausible for an individual who has committed, witnessed, or been the victim of a moral transgression to experience unwanted thoughts or other intrusive symptoms, wish to avoid reminders of the transgression, and feel angry and isolated (11, 37). These are all symptoms associated with PTSD, but may, under the DPM, better characterize a moral injury when considered in the context of additional symptom domains. Further, while it may be argued that intrusive symptoms are less likely to present in the context of a PMIE not also accompanied by a surge of neuroendocrine activity and associated disruptions in fear and memory processing of the sort expected in near-death or other terrifying events, some PMIEs may indeed fit this characterization. Think of a drone pilot who follows commands to bomb a target and later learns he has mistakenly killed a wedding party of women and children. This pilot may experience intrusive thoughts or nightmares, depressed mood, and even increased heart rate when returning to his work station…but are these symptoms better characterized by PTSD or a perceived moral transgression leading to moral failure and moral injury? In the DPM, careful examination of the nature of an individual’s psychological symptoms and associated narratives, in conjunction with analysis of their role appraisal and event-exposure, is required to understand whether psychological symptoms best support a diagnosis of PTSD, moral injury, or both.

#### Social domain

Deficits in social functioning, including interpersonal conflict, social anxiety and avoidance behavior, difficulty building and maintaining relationships, and occupational problems, are core characteristics of traumatic illnesses like PTSD (38). Similarly, in the case of moral injury and particularly in military populations, researchers have observed social disturbances associated with PMIEs and moral injury outcomes, including feelings of social isolation and rejection (20), loss of trust in authority (21), and lack of perceived social support (23). But the social process domain is critically important in any account of moral injury beyond downstream consequences of PMIEs because the moral values that must be transgressed to produce a PMIE are themselves socially derived and maintained. In their review of the social psychology of morality, Ellemers et al. (39) refer to moral values as “socially anchored,” emerging from communal beliefs that define the boundaries of acceptable behavior. Thus, in critical ways, moral injury is a social experience; it is only possible when social (e.g., moral) values are transgressed and where social harm has been done. Whether through perpetration, victimization, or witnessing, an individual who experiences a PMIE may find themselves unmoored without the social anchor that previously secured their sense of right and wrong. In the perpetrator or witness role, an individual who commits or fails to stop a moral transgression that violates salient social boundaries may feel unworthy, ashamed, afraid of the social consequences of discovery. In the victim or witness role, an individual may feel rage or disgust at a moral transgression, especially when perpetrated in an institutional context undergirded by the moral values which have themselves been transgressed, as in the case of a military service member who witnesses the killing of innocents or a devout young Catholic who witnesses abuse or is themselves abused by a priest. In these cases, the consequences of the transgression are grave, but part of what has been shaken is faith in the social prescription of morality. The core feature of the social domain is thus the clash between important social rules or values and actual behavior. In the DPM, careful exploration of the roots of social problems, their temporal links to PMIEs or traumatic experiences, and integration with other symptom and process domains, facilitate development of a holistic account of individual suffering best characterized by traumatic illness, moral injury, or both.

#### Spiritual and religious domain

Spirituality and religion in the context of traumatic illness and moral injury remain fertile territory for investigation. While philosophers and scholars continue to debate the contours of these constructs, for our purposes religion can be understood as a socio-cultural system of beliefs, practices, and norms that structure human interaction and provide both a framework of meaning for everyday experience and answers to metaphysical quandaries, typically in dependence on a god, gods, or other manifestation of the divine (40, 41). Spirituality is less well defined, but can be understood to refer to the human experience of meaning, purpose, and connection with the self, others, nature, the world, and even the totality of existence (42, 43). Much work examining connections between religion, spirituality, and moral injury derives from the military context and is concerned with the role of military chaplains caring for military service members, but clinicians increasingly recognize the utility of a bio-psycho-social-spiritual model for understanding human problems more broadly (42–44). Pew research data (45) show that 48% of Americans identify as religious and spiritual, 27% identify as spiritual but not religious, and 6% identify as religious but not spiritual, while only 18% identify as neither religious nor spiritual. Thus, the great majority of Americans understand their experiences of connection, meaning, and purpose to be integrated within larger religious and/or spiritual structures.

The previous symptom domains we reviewed fit within a relatively well-defined clinical conceptualization of traumatic illness and suggest a related moral injury syndrome, also defined in clinical terms, might be amenable to change through clinical tools like habituation, cognitive restructuring, and pharmacotherapy (46). The religious and spiritual domain marks a departure from this formula. When an individual with a spiritual or religious identity experiences a PMIE, the religious framework that renders the world intelligible and/or the spiritual beliefs and values that lend meaning and structure to experience can be profoundly damaged. The more deeply religious and spiritual constructs are integrated into the individual’s worldview, the more likely the individual is to experience their moral suffering in these terms. Individuals whose worldviews and moral codes are structured by religious or spiritual frameworks, concepts, and language may experience PMIEs and subsequent symptoms through spiritual and religious lenses and describe these experiences using the idioms and metaphors of their religious and spiritual traditions (22). Indeed, a recent latent class analysis of warzone veterans by Currier et al. (47) identified two subgroups of those with moral injury: one subgroup whose experiences were better characterized by psychological symptoms (e.g., self-doubt) and another better characterized by spiritual struggles (e.g., with the divine). Thus, under the DPM, the emergence of religious or spiritual crisis following a salient event-exposure is a strong indicator that a moral injury has occurred. Consistent with the overarching conceptual claims of the DPM, we argue that individuals whose experiences of suffering is mediated by religious and spiritual frameworks may do better with an intervention approach that centers and is informed by these frameworks, rather than one derived from the classical clinical model for treating traumatic illness as a disruption in fear and memory that requires habituation. In other words, if the harm of moral injury is experienced in spiritual and religious terms rather than in psychological or biological terms, the tools of religion and spirituality may be important to consider when developing an intervention approach for moral injury (48). The emerging literature addressing moral injury in the context of military chaplain’s spiritual helping provides support for this view (42, 49).

#### Values, character and identity domain

Many conceptualizations of moral injury remain rooted in clinical language because investigations of moral injury, its definition, and identifying characteristics, emerged from accounts of military veterans diagnosed with traumatic injury (11, 16). More recently, clinicians and researchers have called for a broadening of this approach to include a spiritual dimension, yielding a bio-psycho-social-spiritual model (49). However, the biological and psychological dimensions often receive the most practical emphasis in treatment contexts, meaning that assessment, diagnosis, and intervention for moral injury is often conducted using medical and psychological tools (9, 17). In addition, the share of Americans who identify as neither religious nor spiritual, while small, is growing (50); these individuals may experience moral suffering tied not to spiritual or religious frameworks but to their own deeply held moral beliefs and identity. The DPM points toward an alternative pathway for understanding moral suffering by building on the emerging literature around spirituality in the context of moral injury and integrating a values, character, and identity domain. In this domain, a moral transgression represents failure to adhere to internal moral values, or those prescribed by an important group or institution, incurring a stain on the individual’s moral character (17).

Aristotle’s *Nicomachean Ethics* (51) continues to inform contemporary philosophical and psychological conceptions of virtue and character [e.g., (52–54)]. In the Aristotelian account, character is constructed over time by actions; an excellent character is forged by actions that reflect deeply held moral virtues like courage, honor, generosity, fairness, and truthfulness (17). In Shay’s *Achilles in Vietnam* (2003), heroic Achilles’ character is undone by the slaying of his lover Patroclus – Achilles is transformed into a raging berserker, killing and desecrating the body of his honorable Trojan enemy Hektor in front of Hektor’s family. In this act, at odds with his values and identity as a paragon of Greek warrior virtue, Achilles’ character is damaged and his identity destabilized. Thus, by character, we refer to an established pattern of alignment between internal values and behavior that facilitates a stable moral identity. While, as we observed earlier, internal values are often socially derived or influenced, they may also exist in opposition to perceived social mores. The defining feature of character in this account is its internal locus (i.e., the relation between the moral self the individual idealizes and the one their behavior reveals) rather than adherence to social rules.

As described previously, individuals can occupy multiple positions in moral failure events (e.g., victims, witnesses, and perpetrators), but regardless of their role or position in the event, moral failure provokes a crisis of character (16). In this crisis, the individual’s character, their moral identity, maintained by the integration of moral values with actions, is damaged, either by the individuals’ own actions (perpetrator), their passive presence when others violate moral values (witness), or when trusted representatives or leaders of the social betray moral values and cause harm. In each of these cases, the values that the individual perceives to be foundational to their character and moral identity are challenged with a discontinuity, provoking discrepancy between the individual’s self-narrative and their actual experience (39). Damage to character and moral identity, like spiritual suffering, can be experienced in many ways. Some of these will resemble symptoms associated with traumatic illness (17). For example, individuals who suffer a crisis of faith and a discontinuity in character and moral identity as a result of committing a moral transgression may experience nightmares, feelings of guilt and shame, social withdrawal, and mood changes similar to those suffering from traumatic illness. But there is a key difference; experiences of suffering in the context of a moral injury may not represent a pathological condition that merits a separate diagnosis and clinical intervention. Instead, under the DPM, moral injury may require an alternative approach to healing characterized by tools and strategies that help to reconstruct moral values, repair character, and integrate experiences of moral suffering into a flexible moral identity.

### Labeling

There is considerable overlap between the diagnostic symptoms of traumatic illnesses like PTSD and symptoms and processes characteristic of moral injury (11, 46). Under the DPM framework, symptoms and processes within biological, psychological, social, spiritual and/or \religious, and character, values, and identity domains may be present in both traumatic illness and moral injury contexts. Further, the DPM posits that moral injury and traumatic illness may exist either independently (i.e., one without the other), or in a comorbid fashion. By examining event-exposure characteristics, role appraisals, and symptom and process domains, the DPM provides a flexible framework for determining whether a label of traumatic illness, moral injury, or both, provides a best fit to the information space. This flexibility allows for the hypothesis that moral injury can be mediated by pathways distinct from those linked to threat or fear-based traumatic illness.

#### Vignettes

The first stage of the DPM examines the characteristics of an event exposure to determine whether it fits the description of a traumatic experience, PMIE, or both. While a clear judgment may not be possible at this stage, exploring the nature of the event itself may yield some insight into the role appraisal and pattern of symptoms and processes that follow. For example, we can imagine an individual involved in a sudden and violent car collision when driving on the freeway. This individual may experience shock, terror, and the fear of death, and understand that they are the victim of a terrible accident, but they may not experience any strong moral emotions or feel that a moral transgression has taken place. On the other hand, we can also imagine an individual whose previously loving spouse abruptly disappears, clears out the family bank account, and abandons them and their young children. In this case, the individual may not feel terror, but they may feel that they are the victim of an awful moral transgression. Finally, we can imagine a young soldier clearing buildings in Fallujah, coming under heavy enemy fire day after day, seeing friends die, functioning at maximum alertness, until 1 day he is confronted by a teenager in an open doorway holding an explosive device. The soldier acts as his training dictates, shooting and killing the teenager, saving his own and his squad’s lives. He may continue to experience hyperarousal and intrusive thoughts after returning home. But later, he may also feel a moral transgression has taken place. Perhaps his own. Perhaps the leaders that put him in that country to begin with have transgressed. Perhaps the men that used that teenager have. Perhaps they all have. The soldier may see himself as perpetrating a moral transgression, but he may also see himself as the witness, or even the victim, of others’ transgressions.

These examples demonstrate that, by exploring the nature of the event-experience itself, the DPM facilitates formulation of preliminary hypotheses about how events and role appraisals lead to symptoms and processes characteristic of traumatic illness, moral injury, or both. The car accident victim may begin to experience nightmares about car crashes and hyperarousal when hearing a car horn or tires squeal. They may stop driving and curtail their social activities to avoid having to be in cars. Where previously they found relief from stress in prayer, this is no longer the case after the accident. With these changes come changes in how they see themselves. Where previously they were independent and capable, now they are fearful, embarrassed, isolated, and depressed. They are someone they do not recognize. Thus, we can see the symptom and process domains unfold; biological processes like stress-response and arousal are dysregulated, and psychological symptoms including avoidance, reexperiencing, and mood changes are prominent. While the accident victim’s social functioning and identity are affected, these effects are secondary to biological and psychological symptoms highly suggestive of traumatic illness.

By contrast, the spouse abandoned by their partner may not experience hyperarousal, but they may have intrusive thoughts about why and how such a thing could have happened. Where previously they might have seen a larger plan at work in their life, secure in their belief that by following the rules good things would happen, they now felt cut loose, overcome by anger at their partner and the world. They had done everything right, followed the rules, and for what? They didn’t trust anyone, and they were scared to try. Picking up the pieces of their life felt impossible. In this case, we can see that psychological symptoms including mood changes and negative cognitions are present, but the integrity of values, identity, and character frameworks that render the world intelligible have been damaged by moral transgression highly suggestive of moral injury.

Finally, the combat veteran may return home and be plagued by nightmares of his experiences in Fallujah. He may be hypervigilant in crowds, unable to relax in social settings. He may have intrusive thoughts about his experiences, particularly when seeing young men who remind him of the teenager he had to kill. As a result, he may try to avoid these situations. But he may also begin to wonder if he has lost something important, ineffable, that he will never regain. He was trained and prepared to kill to defend his country, but he didn’t think he’d have to kill a child. He followed the news and learned that there were no weapons of mass destruction hidden in Iraq, and that he had thus been sent to kill and see his friends die on a false pretext. His belief in the moral authority of his leaders was shattered. What had he killed for? What had his friends died for? Here, we can see evidence of both biological and psychological symptoms indicative of traumatic illness, and spiritual and values, character and identity symptoms indicative of moral injury symptoms at work. If referred to the VA, it is likely that this combat veteran would receive a PTSD diagnosis and under the best case circumstances be treated with evidenced-based interventions like Cognitive Processing Therapy [CPT; (8)] or Prolonged Exposure [PE; (55)]. Through the process of exposure and habituation, these approaches may indeed help the combat veteran to resolve his reexperiencing, hyperarousal, and avoidance symptoms. But how can they help with his moral suffering? How can they rebuild the shattered system of values and meaning that undergirded his character and identity? How can they resolve his anger at the politicians who sent him to fight for a lie and his fear that, even though he had to kill to survive, his soul is stained indelibly?

### Intervention approaches

There are well validated and widely used clinical interventions to treat PTSD. Cognitive CPT, PE, and Eye Movement Desensitization and Reprocessing [EMDR; (56)] are the most widely used, and are considered by many to represent the “gold standard” for the treatment of PTSD. In general, these therapies work well for many individuals, yet upward of one half of those receiving treatment with one of these interventions fail to respond; for those suffering from PTSD related to combat, nearly two-thirds still have a diagnosis after completing CPT or PE treatment (57), while EMDR has been shown to be ineffective for treating PTSD in a military population and is recommended only as a last resort (58). While there are numerous explanations for the ineffectiveness of these psychotherapy interventions, ranging from poor adherence in delivering the treatment protocol to lack of organizational support for the implementation of these evidence-based interventions, we believe that a more likely explanation in many cases is that the intervention is not targeting the right set of symptoms.

While existing psychotherapies are reasonably effective in ameliorating the symptoms associated with PTSD, they are less effective in addressing the symptoms associated with moral injury. Specifically, the existing treatments for PTSD fail to provide significant benefit to those suffering from a moral injury which challenges one’s character and identity. Experiencing a violation of a deeply held moral beliefs can result in one questioning their own identity and sense of self, including views that the world is unfair and unjust. Such disillusionment may result in downstream mental and behavioral health problems associated with PTSD, including intense feelings of thwarted belongingness and a disconnection from others, isolation, alcohol and substance misuse and feelings one does not deserve to live. But, it would be unreasonable for an intervention aimed at treating PTSD to resolve these symptoms when they are the result of a moral injury. Instead, interventions are needed that address the rebuilding of one’s damaged identity and character. The key components of such an intervention approach is discussed in the following section.

#### Character development and repair

We argue that when an individual’s experience of suffering is driven primarily by symptoms and processes located within spiritual and/or religious and values, character, and identity domains, and thus indicative of moral injury, the starting point for repair is character. We will briefly review how character is formed and shaped and then discuss how to address rebuilding character in the aftermath of moral injury. Our line of thinking on character will be guided once more by Aristotelian ethics [NE; (51)] followed by tenets of Stoic philosophy as well as other group/institution-based mechanisms for rebuilding character.

##### Character development

In describing human goodness, Aristotle coined the term *ethike arete* or excellence in character that is rooted in the moral values (e.g., courage, friendliness, generosity). Individuals come to know what it means to be good or to do good to themselves and others from the groups they belong to, ranging from their family to their peer groups to formal institutions (e.g., school, church, military). People acquire moral values as a function of both formal learning and informal socialization with in-group others. Over time, these moral values become the ethical markings of character and serve the dual function of defining an individual (e.g., courageous) and prescribing appropriate behavior (e.g., courage). The overarching goal of living life guided by these moral values is *eudaimonia*, a thriving or flourishing life.

##### Character repair

By experience, however, individuals learn that they will often fail to live up to moral values, either by choice or circumstance. In these instances, there exists a dissonance, or a discrepancy between values and behaviors. How then does an individual reconcile this discrepancy? Because social groups are the arbiters of moral values, what mechanisms do these groups have that will allow an individual to preserve their character and repair damage to moral identity?

#### Stoicism: Self-assessment and self-forgiveness

Often described as a philosophy born out of adversity, Stoicism was highly attuned to the causes and consequences of human suffering and was very practical in its approach to *eudaimonia.* Briefly, Stoicism is an Hellenistic philosophy rooted in virtue ethics and pragmatic ideas for leading a virtuous life (59). We propose that one way to rebuild character is to borrow from the Stoics’ practices of self-reflection and self-forgiveness. The Stoics were aware that self-improvement required regular self-assessments. For example, Marcus Aurelius’ *Meditations* was a compilation of his own notes written to himself alone (i.e., not for public consumption) for the purpose of self-reflection and improvement, with some of the text providing details on how he practiced Stoicism. From Seneca, we see more of this self-assessment and self-forgiveness practice: “When the lamp has been removed from my sight, and my wife, no stranger now to my habit, has fallen silent, I examine the whole of my day and retrace my actions and words; I hide nothing from myself, pass over nothing. For why should I be afraid of any of my mistakes, when I can say: ‘Beware of doing that again, and this time I pardon you”’ (60). By examining these writings, we can see that the Stoics understood moral identity and character to be inherently imperfect, in need of examination and care, particularly when confronted with challenging experiences.

Self-forgiveness research has indeed demonstrated empirical support for the practices of character examination, social engagement and accountability, and commitment to change. Woodyatt (in press) recommends the following practical steps in self-forgiveness: (1) understanding the proper role of emotions to avoid self-condemnation, (2) being surrounded by a community that encourages humility and authenticity, and (3) reaffirming the violated values or giving oneself another chance to do better. The latter point is critical for character redevelopment because opportunities to engage in doing good for self and others reinforces the awareness of and capacity for goodness; doing affects being.

ADM James Stockdale, the highest-ranking POW in the Hanoi Hilton, credits stoicism as having helped him endure almost 8 years of torture in prison. In *Courage Under Fire* (1993), he details how forgiveness (of self and others) as well as actively taking part in the “tap code” network for social support, were critical in maintaining and repairing their own and each other’s integrity of character.

#### Religion: Repentance and restoration

Perhaps more than any other social structure, religious institutions have provided ways and means by which an individual can rebuild their character. Koenig and Al Zaben (61) conducted a recent review of religious rituals or spiritual practices used to treat moral injury. For our purposes, we will focus on those that have implications for rebuilding character. Of the eight interventions specified by Koenig and Al Zaben (61), only two appear to have a direct bearing on character. The Pastoral Narrative Disclosure (PND), developed by Carey and Hodgson (49) comprise eight steps, namely: rapport, reflection, review, reconstruction, restoration, ritual, renewal, and reconnection. Underlying these steps is the sacrament of penance, a religious ritual used to absolve wrongdoing and achieve forgiveness and cleansing. PND, however, was designed as an adjunctive, rather than a stand-alone, treatment of moral injury, similar to Litz et al. (62) Adaptive Disclosure Therapy. Analogous to PND, but shorter in length, is Moral Injury Reconciliation Therapy [MIRT; (63)]. The five-session MIRT addresses recognition of moral injury, lament and confession, response using one’s own value system, forgiveness and identity, and reconciliation through habit training. Both these spiritual/religious interventions address the core issue of rebuilding an individual’s character through reconstruction (for PND) and identity and habit training (for MIRT).

## Conclusion

The DPM provides a flexible analytical framework for evaluating event-exposures, role appraisals, and downstream symptoms and processes to facilitate appropriate labeling of complex and heterogenous experiences consistent with traumatic illness, moral injury, or both, with important implications for developing treatment options. While the most thoroughly reviewed evidence-based treatments for traumatic stress hinge on exposure and habituation mechanisms to manage dysregulation of fear and memory systems, these mechanisms often do not address core domains of moral injury identified in the DPM, including spiritual, religious, values, character, and identity domains. This view is consistent with evidence (64) showing that military veterans diagnosed with and treated for PTSD often demonstrate relatively poor outcomes; we argue that these cases may reflect comorbid or discrete moral injury. Further, we argue that because moral injury reflects profound damage to values, character, and moral identity, healing from moral injury will require tools and methods that center these domains.

## Author contributions

NB conceptualized, wrote, and edited the manuscript. HA, SS, and CC contributed to writing and editing the manuscript. All authors contributed to the article and approved the submitted version.

## References

[1] American Psychiatric Association. *Diagnostic and Statistical Manual of Mental Disorders.* 5th ed. Virginia: American Psychiatric Association (2013). 10.1176/appi.books.9780890425596

[2] Hoge CW Castro CA Messer SC McGurk D Cotting DI Koffman RL. Combat duty in Iraq and Afghanistan, mental health problems, and barriers to care. *New Engl J Med.* (2004) 351:13–22. 10.1056/NEJMoa040603 15229303

[3] Castro CA McGurk D. The intensity of combat and behavioral health status. *Traumatology.* (2007) 13:6–23. 10.1177/1534765607309950

[4] Brewin CR Holmes EA. Psychological theories of posttraumatic stress disorder. *Clin Psychol. Rev.* (2003) 23:339–76. 10.1016/S0272-7358(03)00033-3 12729677

[5] Clark DM Ehlers A. Posttraumatic stress disorder: from cognitive theory to therapy. In: Leahy RL editor. *Contemporary Cognitive Therapy: Theory, Research, and Practice.* New York, NY: The Guilford Press (2004). p. 141–60.

[6] Foa EB Huppert JD Cahill SP. Emotional processing theory: an update. In: Rothbaum BO editor. *Pathological anxiety: Emotional Processing in Etiology and Treatment.* New York, NY: The Guilford Press (2006). p. 3–24.

[7] Rauch S Foa E. Emotional processing theory (EPT) and exposure therapy for PTSD. *J Contemp Psychother.* (2006) 36:61–5. 10.1007/s10879-006-9008-y

[8] Resick PA Monson CM Chard KM. *Cognitive Processing Therapy for PTSD: A Comprehensive Manual.* New York NY: Guilford Publications (2016).

[9] Griffin BJ Purcell N Burkman K Litz BT Bryan CJ Schmitz M Moral injury: an integrative review. *J Traumat Stress.* (2019) 32:350–62. 10.1002/jts.22362 30688367

[10] Shay J. *Achilles in Vietnam: Combat Trauma and the Undoing of Character. 1994.* New York, NY: Scribner (2003).

[11] Litz BT Stein N Delaney E Lebowitz L Nash WP Silva C Moral injury and moral repair in war veterans: a preliminary model and intervention strategy. *Clin Psychol Rev.* (2009) 29:695–706. 10.1016/j.cpr.2009.07.003 19683376

[12] Litz BT Kerig PK. Introduction to the special issue on moral injury: conceptual challenges, methodological issues, and clinical applications. *J Traumat Stress.* (2019) 32:341–9. 10.1002/jts.22405 31162737

[13] Nash WP Marino Carper TL Mills MA Au T Goldsmith A Litz BT. Psychometric evaluation of the moral injury events scale. *Mil Med.* (2013) 178:646–52. 10.7205/MILMED-D-13-00017 23756071

[14] Shay J. Moral injury. *Psychoanal Psychol.* (2014) 31:182. 10.1037/a0036090

[15] Litz BT Stein N Delaney E Lebowitz L Nash WP Silva C Moral injury and moral repair in war veterans: A preliminary model and intervention strategy. *Clini Psychol Rev.* (2009) 29:695–706. 10.1016/j.cpr.2009.07.003 19683376

[16] Shay J. *Achilles in Vietnam: Combat trauma and the Undoing of Character.* New York, NY: Simon & Schuster (2003).

[17] Atuel HR Barr N Jones E Greenberg N Williamson V Schumacher MR Understanding moral injury from a character domain perspective. *J Theoret Philos Psychol.* (2021) 41:155–73. 10.1037/teo0000161

[18] Koenig HG Ames D Youssef NA Oliver JP Volk F Teng EJ The moral injury symptom scale-military version. *J Relig Health.* (2018) 57:249–65. 10.1007/s10943-017-0531-9 29196962

[19] Williamson V Stevelink SA Greenberg N. Occupational moral injury and mental health: systematic review and meta-analysis. *Br J Psychiatry.* (2018) 212:339–46. 10.1192/bjp.2018.55 29786495

[20] Flipse Vargas A Hanson T Kraus D Drescher K Foy D. Moral injury themes in combat veterans’ narrative responses from the National Vietnam Veterans’ Readjustment Study. *Traumatology.* (2013) 19:243–50. 10.1177/1534765613476099

[21] McCormack L Ell L. Complex psychosocial distress postdeployment in veterans: reintegration identity disruption and challenged moral integrity. *Traumatology.* (2017) 23:240. 10.1037/trm0000107

[22] Wortmann JH Eisen E Hundert C Jordan AH Smith MW Nash WP Spiritual features of war-related moral injury: a primer for clinicians. *Spiritual Clin Pract.* (2017) 4:249. 10.1037/scp0000140

[23] Currier JM Farnsworth JK Drescher KD McDermott RC Sims BM Albright DL. Development and evaluation of the expressions of moral injury scale—military version. *Clin Psychol Psychother.* (2018) 25:474–88. 29282787 10.1002/cpp.2170

[24] Shay J. Moral injury. *Psychoana Psychol.* (2014) 31:182.

[25] Ehlers A Mayou RA Bryant B. Cognitive predictors of posttraumatic stress disorder in children: results of a prospective longitudinal study. *Behav Res Therapy.* (2003) 41:1–10. 10.1016/S0005-7967(01)00126-7 12488116

[26] Halligan SL Michael T Clark DM Ehlers A. Posttraumatic stress disorder following assault: the role of cognitive processing, trauma memory, and appraisals. *J Consult Clin Psychol.* (2003) 71:419. 10.1037/0022-006X.71.3.419 12795567

[27] Marsac ML Ciesla J Barakat LP Hildenbrand AK Delahanty DL Widaman K The role of appraisals and coping in predicting posttraumatic stress following pediatric injury. *Psychol Trauma.* (2016) 8:495–503. 10.1037/tra0000116 27065067 PMC4925202

[28] Yehuda R. Biology of posttraumatic stress disorder. *J Clin Psychiatry.* (2001) 62:41–6. 11495096

[29] Yehuda R Koenen KC Galea S Flory JD. The role of genes in defining a molecular biology of PTSD. *Disease Mark.* (2011) 30:67–76. 10.1155/2011/185354 PMC3825239 21508511

[30] Amihaesei IC Mungiu OC. Posttraumatic stress disorder: neuroendocrine and pharmacotherapeutic approach. *Med Surg J.* (2012) 116:563–6. 23077954

[31] Pitman RK Rasmusson AM Koenen KC Shin LM Orr SP Gilbertson MW Biological studies of post-traumatic stress disorder. *Nat Rev Neurosci.* (2012) 13:769–87. 10.1038/nrn3339 23047775 PMC4951157

[32] Polimanti R Wendt FR. Posttraumatic stress disorder: from gene discovery to disease biology. *Psychol Med.* (2021) 51:2178–88. 10.1017/S0033291721000210 33583458 PMC8364573

[33] Meaney M Yehuda R. Epigenetic mechanisms and the risk for PTSD. In: Nemeroff C Marmar C editors. *Post-Traumatic Stress Disorder.* Oxford: Oxford University Press (2018). p. 293–314. 10.1093/med/9780190259440.003.0017

[34] Farnsworth JK Drescher KD Evans W Walser RD. A functional approach to understanding and treating military-related moral injury. *J Context Behav Sci.* (2017) 6:391–7. 10.1016/j.jcbs.2017.07.003

[35] Galatzer-Levy IR Bryant RA. 636,120 ways to have posttraumatic stress disorder. *Perspect Psychol Sci.* (2013) 8:651–62. 10.1177/1745691613504115 26173229

[36] Olbert CM Gala GJ Tupler LA. Quantifying heterogeneity attributable to polythetic diagnostic criteria: theoretical framework and empirical application. *J Abnormal Psychol.* (2014) 123:452. 10.1037/a0036068 24886017

[37] Currier JM Holland JM Malott J. Moral injury, meaning making, and mental health in returning veterans. *J Clin Psychol.* (2015) 71(3):229–40. 25331653 10.1002/jclp.22134

[38] Frueh BC Turner SM Beidel DC Cahill SP. Assessment of social functioning in combat veterans with PTSD. *Aggress Violent Behav.* (2001) 6:79–90. 10.1016/S1359-1789(99)00012-9

[39] Ellemers N Van Der Toorn J Paunov Y Van Leeuwen T. The psychology of morality: a review and analysis of empirical studies published from 1940 through 2017. *Pers Soc Psychol Rev.* (2019) 23:332–66. 10.1177/1088868318811759 30658545 PMC6791030

[40] Spiro ME. Religion: problems of definition and explanation. *Anthropol Approaches study Relig.* (1966) 85:96.

[41] Merriam-Webster.com. (2022). Available online at: https://www.merriamwebster.com/dictionary/religion (accessed February 15, 2022).

[42] Hodgson TJ Carey LB. Moral injury and definitional clarity: betrayal, spirituality and the role of chaplains. *J Relig Health* (2017) 56:1212–28. 10.1007/s10943-017-0407-z 28526912

[43] Puchalski CM. Physicians and patients’ spirituality: ethical concerns and boundaries in spirituality and health. *AMA J Ethics.* (2009) 11:804–15. 10.1001/virtualmentor.2009.11.10.oped1-0910 23206948

[44] Shay J. *Achilles in Vietnam: Combat Trauma and the Undoing of Character. 1994.* New York, NY: Scribner (2003).

[45] Lipka M Gecewicz C. *More Americans now say they’re Spiritual but not Religious.* Washington, D.C: Pew Research Center (2017).

[46] Jinkerson JD. Defining and assessing moral injury: a syndrome perspective. *Traumatology.* (2016) 22:122. 10.1037/trm0000069

[47] Currier JM Isaak SL McDermott RC. Validation of the expressions of moral injury scale-military version-short form. *Clin Psychol Psychother.* (2020) 27:61–8. 10.1002/cpp.2407 31657075

[48] Norma S Maguen S. *Moral Injury. National Center for PTSD, U.S. Department of Veterans Affairs.* (2021). Available online at: https://www.ptsd.va.gov/professional/treat/cooccurring/moral_injury.asp (accessed Feb 15, 2022)

[49] Carey LB Hodgson TJ. Chaplaincy, spiritual care and moral injury: Considerations regarding screening and treatment. *Front Psychiatry.* (2018) 9:619. 10.3389/fpsyt.2018.00619 30568605 PMC6290645

[50] Lipka M. *10 facts about atheists.* Washington, D.C: Pew Research Center (2019).

[51] Ameriks K Clarke DM. *Aristotle: Nicomachean Ethics.* Cambridge: Cambridge University Press (2000).

[52] Haidt J. The new synthesis in moral psychology. *Science* (2007) 316:998–1002. 17510357 10.1126/science.1137651

[53] Lapsley DK Power F. *Character Psychology And Character Education.* Notre Dame, IND: University of Notre Dame Press (2005).

[54] Peterson C Seligman ME. *Character Strengths And Virtues: A Handbook And Classification.* (Vol. 1). Oxford: Oxford University Press (2004).

[55] Foa EB. Prolonged exposure therapy: past, present, and future. *Depress Anxiety.* (2011) 28:1043–7. 10.1002/da.20907 22134957

[56] Shapiro F. *Eye Movement Desensitization and Reprocessing (EMDR) Therapy: Basic Principles, Protocols, and Procedures.* New York, NY: Guilford Publications (2017).

[57] Steenkamp MM Litz BT Hoge CW Marmar CR. Psychotherapy for military-related PTSD: A review of randomized clinical trials. *JAMA.* (2015) 314:489–500. 10.1001/jama.2015.8370 26241600

[58] Verstrael S van der Wurff P Vermetten E. Eye movement desensitization and reprocessing (EMDR) as treatment for combat-related PTSD: a meta-analysis. *Mil Behav Health.* (2013) 1:68–73. 10.1080/21635781.2013.827088

[59] Pigliucci M. *How To Be A Stoic: Using Ancient Philosophy To Live A Modern Life.* New York, NY: Basic Books (2017).

[60] Seneca LA Davie J Reinhardt T. *Dialogues And Essays.* Oxford: Oxford University Press (2008).

[61] Koenig HG Al Zaben F. Psychometric validation and translation of religious and spiritual measures. *J Relig Health.* (2021) 60:3467–83. 10.1007/s10943-021-01373-9 34331172

[62] Litz BT Lebowitz L Gray MJ Nash WP. *Adaptive Disclosure: A New Treatment for Military Trauma, Loss, and Moral Injury.* New York, NY: Guilford Publications (2017).

[63] Lee LJ. *Moral Injury Reconciliation: A Practitioner’s Guide for Treating Moral Injury, PTSD, Grief, and Military Sexual Trauma through Spiritual Formation Strategies.* London: Jessica Kingsley Publishers (2018).

[64] Steenkamp MM Litz BT Marmar CR. First-line psychotherapies for military-related PTSD. *JAMA* (2020) 323:656–7. 10.1001/jama.2019.20825 31999301

